# Glimpsing the Future of Animal Welfare through a Bottle of Milk: Insights from Chinese University Students

**DOI:** 10.3390/foods12214044

**Published:** 2023-11-06

**Authors:** Yaoming Liang, Ruiqi Chen, Hongfu Liu, Li Han, Shu Yin

**Affiliations:** 1 College of Veterinary Medicine, South China Agricultural University, Guangzhou 510642, China; evanliang@scau.edu.cn; 2 College of Economics & Management, South China Agricultural University, Guangzhou 510642, China; ricky_ccc@163.com (R.C.); lhf1192896646@163.com (H.L.); 3 College of Public Management, South China Agricultural University, Guangzhou 510642, China; 4 Student Affairs Department (Graduate Student Affairs Department) of the Party Committee, South China Agricultural University, Guangzhou 510642, China

**Keywords:** animal welfare, consumption preferences, university students, milk, discrete choice experiment

## Abstract

The consumption patterns of university students hold the power to significantly influence market trends. This study illuminates the escalating emphasis on animal welfare in these students’ purchasing choices, specifically concerning milk products. Utilizing a discrete choice experiment, we identified a pronounced preference among students for milk products with animal welfare certifications. Students were segmented into three categories based on their motivations: “Quality–Oriented” (20.55%), “Emotionally Intuitive” (30.67%), and “Quality–Emotion Balanced” (48.77%). The “Emotionally Intuitive” group manifested the most robust inclination toward such certifications. Based on these findings, we recommend tailored market strategies targeting these distinct segments. Moreover, our findings emphasize the importance of intensifying animal welfare education, shaping a market aligned with animal welfare principles, and fostering a broader societal environment attuned to animal welfare.

## 1. Introduction

In the current global context, animal welfare is more than just an ethical concern; it has become a symbol of societal progress and cultural evolution [1,2]. Ensuring better living standards for animals is linked not only to their rights but also to environmental conservation, human health, and food security. Global trends in food choices reflect the increasing awareness regarding the sources of our food and the ethics behind its production. The dairy industry, in particular, has been under the lens, with consumers globally showing heightened interest in the welfare of dairy animals. Factors such as the humane treatment of animals, the environmental impact of dairy farming, and health implications have taken center stage in influencing consumer choices [3]. In response to significant global socioeconomic changes, many countries are increasingly recognizing the importance of animal welfare [4]. China, undergoing significant sociocultural and economic changes, is emphasizing its dedication to this issue. This growing awareness is evident in consumer behavior, especially among younger generations, with an increased preference for products that focus on animal welfare [5].

In China, factors like rapid urbanization, increased globalization, and a growing emphasis on health and the environment have changed consumer behaviors. Chinese consumers, particularly of the younger generation, increasingly want to know the origins of their food and the ethics of its production. For dairy products such as milk, brand reputation regarding animal welfare, perceived health advantages, and product authenticity are major determinants in their purchasing decisions [6]. Recent research in animal welfare has focused on products associated with animal welfare [7,8,9]. Studies have shown a clear preference among consumers for products labeled as animal-friendly [10,11,12]. In the context of dairy, decisions often hinge on the perceived integrity of the product, with animal welfare being a significant consideration [3].

It is worth noting that in China, milk is not just viewed as a drink but often considered a symbol of nutrition and health, especially for the younger generation. This sentiment magnifies the importance of animal welfare in dairy purchases. If consumers believe that the animals were treated well, they implicitly trust the quality and health benefits of the derived milk [13]. However, there remains a gap in understanding the consumption patterns of Chinese university students. These students, as representatives of the younger generation and its mindset, are likely to become key supporters and promoters of animal welfare [14,15]. Investigating their awareness, inclinations, and consumption patterns can provide valuable insights into regional tendencies and potential shifts in animal welfare perceptions.

Milk, a staple in many university students’ diets, is directly linked to the welfare of dairy cattle. With numerous dairy brands in the market, students’ choices often reflect their awareness and concern for animal welfare. Given the role of university students as a reflection of societal values and their position as future primary consumers [15], it is crucial to understand their preferences regarding dairy products. Hence, our study primarily focuses on understanding the consumption behaviors and attitudes of Chinese university students toward milk products endorsed for animal welfare. By doing this, we intend to identify the primary drivers behind their purchasing decisions and understand the potential implications for the livestock industry at large.

In light of this, the following hypotheses are proposed in our study:

(1) Chinese university students have a distinct preference for milk products emphasizing animal welfare;
(2) Factors such as the product’s perceived integrity, brand reputation, and associated health benefits significantly influence their purchasing decisions.

We will explore these consumption preferences in depth through choice experiments. Our goal is to uncover the underlying factors influencing this group’s buying behaviors, thereby providing a detailed analysis of their purchase patterns. These insights can be invaluable for the livestock industry, offering crucial market data to inform future strategies and initiatives.

Our research provides key contributions to the academic field. By examining the specific demographic of university students, we present a detailed consumer profile to both livestock and marketing sectors. This thorough study offers strategic insights, helping to optimize product positioning and clarify brand narratives. More than merely recording consumer trends, our research delves into the underlying psychological drivers of these preferences, offering marketers a richer, more informed viewpoint. This insight is valuable in addressing consumer demands more effectively. While our study primarily focuses on the Chinese context, particularly the Guangdong Province, the shifts in China’s stance on animal welfare likely resonate in other emerging markets. Such countries can gain insights from China’s path, adapting strategies that align with their unique cultural and market scenarios, thus furthering the global commitment to animal welfare.

## 2. Materials and Methods

### 2.1. Discrete Choice Experiment Design

The discrete choice experiment (DCE) provides a methodological framework to evaluate consumers’ preferences and determine their willingness to pay [16]. In the domain of milk consumption, several factors influence consumer decisions, including conventional quality criteria like color, aroma, protein content, nutritional profile, safety, appearance, and convenience [17,18]. Additionally, aspects of ecological ethics, notably animal welfare and environmental conservation, also play a role [19,20]. Within a DCE, participants make choices from a set of product or service options, distinguished by various attributes and their levels [13,21]. These choices allow researchers to ascertain the relative importance of specific attributes and levels. The introduction of a monetary attribute facilitates quantification of the willingness to pay for different options [10]. For this investigation, the DCE approach is utilized to explore Chinese university students’ preferences for milk products with an emphasis on animal welfare. 

The selection of attributes and their corresponding levels is a crucial step in the design of any discrete choice experiment. These choices should be both theoretically meaningful and relevant to the context of the study. Our process in determining these attributes spanned two primary phases: 

Phase 1. Our initial step was to survey various bottled milk brands available at campus supermarkets. Through a careful examination of the packaging, we cataloged a range of attributes, such as price, brand, shelf life, production date, nutrient content (emphasizing levels of protein, calcium, and fat), milk source, preservation method, packaging design, sterilization method, and other specific certifications or labels that were prominently displayed.

Phase 2. Seeking empirical validation of our observations, we undertook a preliminary survey with 41 participants to ascertain the primary attributes they consider while purchasing milk. The results affirmed our observations from Phase 1 in addition to providing a quantifiable measure of their significance. Here are the salient findings: price (63.41%), brand (60.98%), freshness, encapsulated by both shelf life (60.98%) and production date (58.54%), nutrient content with specifics on protein (48.78%), calcium (41.46%), and fat (31.71%), milk source (29.27%), preservation method (29.27%), packaging (24.39%), sterilization method (14.63%), and others (0%).

After these primary phases, we prioritized our attributes for the study. We retained the top four attributes based on their salience from our survey results and introduced an additional “certification or label” category. Notably, while our market survey revealed a scarcity of milk products with specific animal welfare labeling, the importance of this criterion in our research necessitated its inclusion. Thus, animal welfare was incorporated as a distinct certification attribute. Concurrently, in recognizing the well-established significance of organic certifications in milk consumption and their widespread acknowledgment in the market, we also integrated “organic certification”. This approach allowed us to compare the significance of animal welfare alongside the prevailing organic certification, ensuring a comprehensive representation of market certifications.

The selection of attributes and their corresponding levels is instrumental in generating meaningful insights from a DCE. Based on the specifics of milk consumption and the prevailing market dynamics, we outline the rationale for our attribute choices, subsequently providing an explanation for the designated levels.

Brand: In the dairy sector, brand name acts as a proxy for a slew of factors, from perceived quality and taste to overall trustworthiness. With the current market composed of both local and foreign players, it is pivotal to distinguish between “Domestic” and “International” brands. This not only reflects consumers’ potential biases toward familiar local brands versus the perceived premium nature of international ones, but also gauges brand loyalty in the face of growing international market penetration.

Label: As consumer conscience veers toward ethical and environmentally responsible choices, labels gain prominence [22]. They not only ensure consumer confidence in product quality and safety but also represent specific ethical standards. The absence or presence of an “Organic” label sheds light on eco-friendly agricultural practices and consumer concern for environmental sustainability. The “Animal Welfare” label, while less commonplace, carries a significant ethical weight. It emphasizes the humane treatment of dairy livestock and showcases a consumer’s active support for animal rights. The inclusion of the “No label” category aids in gauging baseline preferences, effectively capturing the weightage consumers assign to these ethical considerations.

Protein Content: A primary motive behind milk consumption is its nutritive value. Protein stands out as a vital metric. Differentiating between protein contents—3.2, 3.6, and 4.0 g per 100 mL—acknowledges the array of choices that the market presents, tapping into health-driven consumer segments that prioritize protein intake.

Shelf Life: The dairy industry, where product freshness can dictate consumption choices, renders shelf life a crucial attribute. Our levels of 1, 3, and 5 months span the continuum of available products, ranging from those demanding imminent consumption to ones assuring extended freshness. This differentiation caters to varying consumer demands, from those prioritizing freshness to those valuing convenience [23].

Price: The price of milk is a composite reflection of factors such as brand, production methods, and perceived quality. By demarcating four distinct price points (CNY 2.8, 4.8, 6.8, and 8.8), we encapsulate the prevalent market pricing spectrum. This gradation permits an exploration of consumer price sensitivity, especially when juxtaposed against other attributes. To determine these price points, we referenced the average prices for 250 mL of pure milk on the major Chinese e-commerce platforms, Taobao and Jingdong. Furthermore, our choice of price points was deliberately influenced by cultural and psychological pricing strategies; the number “8” is considered auspicious in Chinese culture, symbolizing wealth due to its pronunciation akin to “fa”, and a price of CNY 8.8, while nearly identical to CNY 9.0, is perceived as significantly more appealing due to this cultural context and the psychological impression of receiving a better deal [24].

Table 1 lists the milk attributes and their levels considered in our study. From these, 216 product or service options can be generated, leading to 23,220 possible combinations. To manage the experiment and avoid potential interactions between factors, we used the Ngene1.2.1 software and the D-optimal fractional factorial design, selecting 36 combinations to assess the importance of animal welfare attributes in milk to university students. To reduce choice overload, we divided these combinations into six groups, each with six choice tasks.

**Figure 1:**
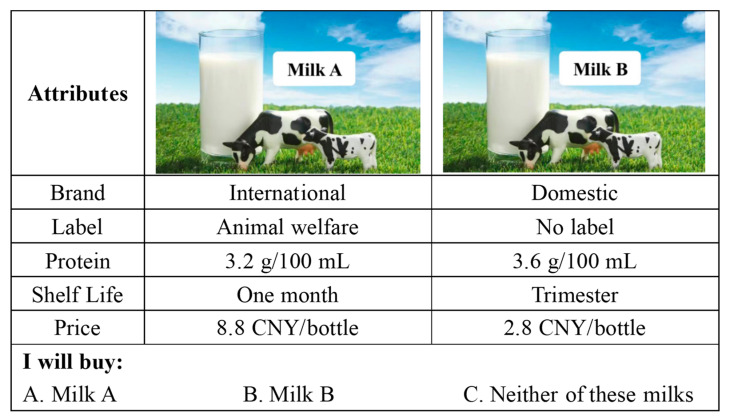
Representative choice set.

Figure 1 provides a sample of the choice sets used in the study. To enhance the realism of the experiment, each set includes two hypothetical alternatives and a “no purchase” option. Participants are given different choice scenarios based on their birth month. For example, those born in January or July are presented with the first set of choice tasks. To minimize potential hypothetical bias, participants are introduced to a “cheap-talk” script before the experiment begins, a method known to reduce biases according to Cummings and Taylor (1999) [25].

### 2.2. Survey Design

Guangdong, with its intertwining of animal welfare concerns and local developmental intricacies shaped by unique cultural influences, emerged as our chosen research setting. As an early frontrunner in China’s economic globalization, Guangdong’s extensive exposure to international cultures and rapid alignment with global standards makes it an intriguing region for exploring evolving concepts like animal welfare.

Its significant GDP of CNY 12.91 trillion in 2022 (approximately USD 1.89 trillion at year-end exchange rates) reflects not only economic strength but also changing consumer preferences. Economic growth often corresponds to an increase in health-conscious lifestyles and a heightened awareness of areas like animal welfare and environmental conservation. Guangdong’s diverse cultural spectrum, from its Cantonese culinary traditions to regional identities such as Guangfu, Chaoshan, and Hakka, creates a complex but cohesive context. This setting offers valuable insights into regional influences on animal welfare perceptions.

We employed “Wenjuanxing”, a leading survey platform in China, and primarily targeted undergraduate and graduate students in Guangdong. Our survey covered multiple dimensions, including respondent demographics, household dynamics, prevalent milk consumption patterns, and a detailed DCE focusing on the monetary valuation of animal welfare in relation to milk quality attributes.

Before the large-scale distribution of our survey, we initiated an exhaustive evaluation process. Our research team conducted the initial review, assessing the survey’s integrity and relevance. We then sought external feedback from a diverse panel of domain experts, including professionals in statistics and econometrics, scholars in consumer behavior, and experts in animal welfare. Their diverse expertise was crucial in refining our instrument. In addition to the academic and professional experts, feedback from undergraduate and postgraduate students ensured that our questions resonated well with the target demographic. Their suggestions primarily revolved around refining the phrasing of questions, ensuring the comprehensive coverage of pertinent information, and optimizing the sequence of the questionnaire sections. Following these meticulous stages of validation, the survey was disseminated in March 2023.

To ensure robust statistical insights and to meet the strict requirements of the DCE, we followed established guidelines related to choice experiment designs in our sample size calculation [26,27]. We divided the 36 choice sets into six clusters, necessitating a minimum of 167 respondents. By the end of our data collection phase in August 2023, we had 1036 responses. Following a detailed data curation process, 978 valid responses remained, leading to a total of 5868 fully realized choice experiments. All statistical analysis was carried out using Stata 17.0 (Stata Corp., College Station, TX, USA, 2021) [28] and R 4.1.2 (R Core Team, Vienna, Austria, 2021) [29]. For a detailed breakdown of the demographic characteristics of our respondents, please refer to Section 2.4.1 and Table 2.

### 2.3. Statistical Analysis

To explore the preferences of college students for milk labeled with animal welfare certifications, we employed a DCE. The design of this DCE is grounded in the principle that when consumers select from multiple alternatives, their choices reflect their intrinsic valuations of the different attributes of the products.

Central to the discussion on consumer choices is the concept of utility. In the random utility model, utility is conceptualized as consisting of a deterministic component (which depends on the attributes of the option) and a random component. Specifically, the utility for individual *n* in context *t* for choosing option *I* from choice set *C* can be represented as

*U* = *V_nit_* + *ε_nit_* (1)

In this framework, if *U_nit_* > *U_njt_* for all *j* not equal to *I*, individual *n* would opt for choice *i*. Building on this, the probability of individual *n* choosing option *I* is

*P_nit_* = *Prob*(*V_nit_* + *ε_nit_* > *V_njt_* + *ε_njt_*) (2)

for all *j* in *C* and *j* not equal to *i*.

For the traditional Logit model, homogeneity in consumer preferences is typically assumed. However, it is well understood that consumer preferences are heterogeneous in practice. To capture this heterogeneity, we adopted the Mixed Logit model, which permits in-sample variation in preferences. In the model, the deterministic part of the utility can be described as

*V_nit_* = *β’χ_nit_* (3)

where *β* is a vector of random parameters representing consumer preferences and *χ_nit_* is a vector of all attributes in choice *i*. According to Train and Sonnier (2003) [30], the probability that individual *n* in scenario *t* selects option *I* from choice set *C* is given as

(4) $$P_{nit}=\int \frac{exp(V_{nit})}{\sum_{j\;}exp(V_{njt})\;}\;f(\beta )d\beta$$

where *f*(.) denotes the distribution of the random parameters. If a parameter is fixed at *β_c_* (i.e., non-random), its distribution collapses, meaning *f*(*β_c_*) approaches infinity, while *f(β)* equals zero elsewhere. 

To articulate and quantify the value assessments by consumers, we turn to the concept of willingness to pay (*WTP*). Based on the model estimates, the *WTP* for a particular attribute can be defined as

*WTP* = *−β_k_*/*β_p_* (5)

where *β_k_* is the estimated coefficient for the *k*^th^ attribute, and *β_p_* is the estimated price coefficient. Given the ordinal nature of this utility, we further employed a parametric bootstrap method to generate 95% confidence intervals for the *WTP* valuations.

In summary, the combined use of choice experiments and the Mixed Logit model provides us with a robust tool to delve into the intrinsic valuations by college students for milk labeled with animal welfare certifications, offering valuable insights for market strategies and food policies.

### 2.4. Sample Description

#### 2.4.1. Demographic Characteristics

Our sample’s core characteristics and consumption behaviors are detailed in Table 2. The surveyed individuals’ average age was 20.82 years, and females were predominant at 59.00%. A significant portion (85.79%) hailed from urban zones in contrast to the 14.21% who were from rural backgrounds. Examining academic qualifications, those pursuing undergraduate and associate degrees made up 85.17%, leaving postgraduates at 14.83%. On average, the monthly expenditures among the respondents amounted to CNY 1732.34.

The ways in which milk was procured underscores the digital shift and need for convenience among university goers: 58.69% leaned toward online buying. Significantly, nearly half (49.18%) of the participants have been consistent milk consumers for more than 11 years, reinforcing the beverage’s continued role in their diets. A glance at the BMI statistics indicates that 65.03% of our sample are within the normal weight range. Those falling into the underweight bracket represent 17.79%, closely followed by the overweight or obese category at 17.18%.

#### 2.4.2. Perceptions of Animal Welfare

In the current investigation, we delved into university students’ perceptions of animal welfare. This was carried out across four distinct dimensions, namely the juxtaposition of life versus welfare, prioritizing human welfare over animal welfare, the economic implications of welfare-focused farming, and discerning between genuine welfare concerns and business objectives. The selection of these particular dimensions was motivated by the ongoing debates and misconceptions surrounding the realm of animal welfare in today’s society.

Participants expressed their views on each dimension using a 5-point Likert scale. A score of “1” corresponds to “strongly disagree” while “5” resonates with “strongly agree”. The outcomes, as presented in Table 3, reveal satisfactory reliability for each dimension, with Cronbach’s alpha values consistently exceeding 0.75. An overall reliability score of 0.85 was achieved, which signals a high degree of reliability in the measurements. When viewed holistically, the sampled university students exhibit a generally affirmative stance toward animal welfare. The majority took issue with the statement that farm animals’ welfare is inconsequential given their eventual fate of being consumed, implying a recognition of the importance of ensuring animal well-being irrespective of their ultimate purpose. An additional insight derived from Table 3 is the students’ perspective on the financial implications of welfare-centric farming. While there was acknowledgment of its associated costs, the majority did not see this as a significant barrier to embracing the concept of animal welfare. Moreover, the relatively lower mean values for some statements suggest a prevalent sentiment among students that genuine reasons drive welfare farming practices beyond sheer commercial or novelty motives.

#### 2.4.3. Factors Influencing the Perception of Animal Welfare Milk

Building upon the foundational work of Wang and Gu (2014) [31], in our study, we meticulously examine university students’ cognitive attitudes toward animal welfare milk. This exploration is bolstered by highlighting two interlinked dimensions: product quality and emotional resonance. Specifically, we sought to understand how students discern tangible attributes like taste, safety, and health benefits (product quality) in juxtaposition with intangible values, such as emotional well-being and ethical considerations (emotional resonance) when they consume animal welfare milk.

To enrich this understanding, an evaluative framework, inspired by Liang et al., (2022) [1], was employed. Notwithstanding their primary focus on meat products, we identified an alignment in attributes relevant to our study. Using a 5-point Likert scale, we solicited participants’ sentiments and beliefs. As illustrated in Table 4, it is conspicuous that the ethical treatment of farm animals stands out as a prime consideration, with a mean score of 3.454. Additionally, the attribute linked to health also gains substantial attention, surpassing a mean score of 3.367. An overarching reliability score of 0.909 adds credence to the validity of our data.

Advancing from individual attributes to holistic orientations, the factors affecting the perception of animal welfare milk can be synthesized into two dominant sub-dimensions:

(1) Product Quality Orientation. This dimension encapsulates perceptions related to the taste, safety, and health benefits of animal welfare milk.
(2) Emotional Resonance. This primarily focuses on the intangible rewards that students experience when consuming animal welfare milk, coupled with their empathetic stance toward the well-being of farm animals.

Students’ alignment with these dimensions was gauged using the mean scores from the product quality (comprising 3 items) and emotional resonance (comprising 2 items) sections. Respondents exhibiting a stronger affinity toward product quality were earmarked as “Quality–Oriented”, while those leaning toward emotional considerations were dubbed “Emotionally Intuitive”. Those straddling both dimensions equally were cataloged under the “Quality–Emotion Balanced” group.

**Figure 2:**
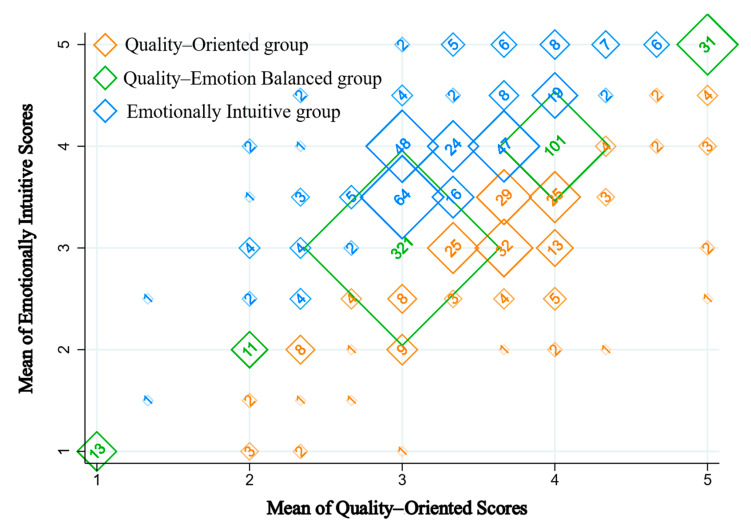
Students’ preferences: quality vs. emotional considerations. The numerical values indicate the total number of university students within each category.

As delineated in Figure 2, a significant cohort, nearly 48.77%, regarded both dimensions—quality and emotion—with equal emphasis. This cohort was followed by the “Emotionally Intuitive” cluster (30.67%) and then the “Quality–Oriented” contingent (20.55%) (percentages may not sum to 100% due to rounding). This stratification suggests that university students navigate a delicate interplay between tangible product quality and intangible emotional considerations, albeit with a minor skew toward the latter.

## 3. Empirical Results and Econometric Analysis

### 3.1. Preferences of University Students toward Animal Welfare Milk

Table 5 delineates the outcomes of the Mixed Logit regression, shedding light on Chinese university students’ inclinations and preferences for specific attributes of animal welfare milk. At a holistic level, students manifest discernible preferences across factors such as brand affiliation, labeling, protein concentration, and product longevity. Notably, the “Organic” attribute emerges as the preeminent driver of value perception, exhibiting a coefficient (*β*) of 1.377. This is closely trailed by the “animal welfare” attribute, with a coefficient of 1.237. In stark contrast, attributes like protein content (*β* = 0.466) and shelf life (*β* = 0.024) seem to hold relatively subdued importance. Intriguingly, the “import” attribute did not achieve statistical significance, alluding to the inference that the origin—whether domestic or international—does not substantially sway students’ predilections when navigating choices in the realm of animal welfare milk.

Further diving into the variance associated with each attribute, we find that, with the exception of “import” and “shelf life”, attributes such as “organic”, “animal welfare”, and “protein content” are significant at the 1% level, reinforcing the validity of employing the Mixed Logit framework for this investigation. Furthermore, the trans-disciplinary perspective reveals a remarkable uniformity in the preferences of students hailing from diverse academic backgrounds like agriculture, science, engineering, and the humanities. This homogeneity suggests a unified stance and understanding among students when it comes to the ascribed value of the animal welfare attributes of milk.

### 3.2. Segmented Analysis of Consumer Motivations behind Animal Welfare Preferences

This study delves into the purchasing patterns of university students when selecting milk products endorsed with animal welfare labels. We concentrate on three distinct cognitive consumer drivers, namely “Quality–Oriented”, “Emotionally Intuitive”, and “Quality–Emotion Balanced”. Our goal is to unravel the key influences shaping their preferences.

According to the regression results shown in Table 6, each consumer category demonstrates a significant predilection for the animal welfare attribute, though to varying degrees. The “Emotionally Intuitive” group exhibits the most pronounced preference for animal welfare (*β* = 1.646). This underscores a heightened ethical awareness regarding animal treatment within this cohort. Their behavior indicates a profound alignment of animal welfare-labeled milk products with their core emotional values. On the other hand, the “Quality–Oriented” group, while valuing animal welfare, assigns supreme significance to the protein content (*β* = 0.580), reflecting their intrinsic emphasis on the nutritional and health characteristics of the product. The “Quality–Emotion Balanced” group represents an equilibrium between the other two groups, appreciating both quality and ethical dimensions. Their purchasing behavior embodies a balanced interplay of rationality and emotion. Notably, a universal endorsement of the organic certification is evident across all consumer categories. This suggests a potential perception among students of a linkage between organic standards and ethical production—a dimension meriting further exploration in subsequent research endeavors.

In summation, the motivations underpinning university students’ inclination for animal welfare-labeled milk products are multifarious. For marketers, it is imperative to comprehend these subtle inclinations. Crafting marketing strategies that mirror these specific motivations will pave the way for more impactful and resonant campaigns tailored to the university student demographic.

### 3.3. Differential Willingness to Pay for Animal Welfare Milk Based on Consumer Motivations

**Figure 3:**
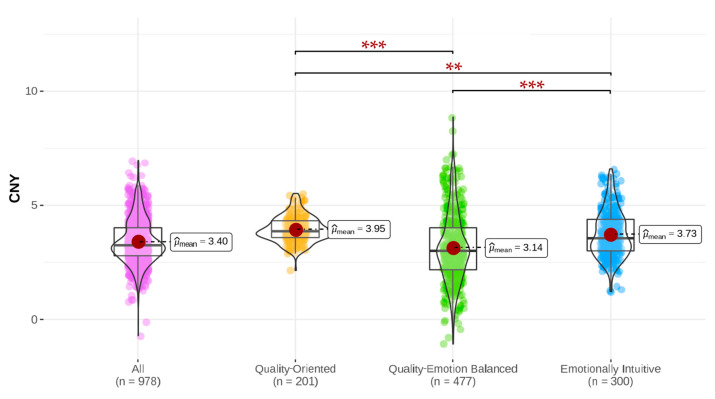
Differential willingness to pay based on consumer motivations. The symbols *** and ** denote significant discrepancies at the 0.1% and 1% confidence intervals, respectively.

To better understand the willingness-to-pay (WTP) dynamics for animal welfare-labeled milk among university students of different motivational categories, we evaluated the price premium they associated with such labeled products. Figure 3 reveals that the student cohort under investigation was willing, on average, to pay an extra CNY 3.40 for milk branded with an animal welfare label, though significant variations were evident among the three consumer motivation categories.

Students with a quality-driven focus recorded the highest WTP, willing to part with an additional CNY 3.95. The narrowness of their standard deviation underscores a consistent belief in the intimate relationship between the quality of milk and the well-being of the animals. This pattern indicates that when consumers discern a tangible link between product excellence and humane animal treatment, their readiness to bear a higher cost strengthens.

Those driven by emotional intuition presented a WTP premium of CNY 3.73, mirroring their ethos of ethically conscious consumption. These students, guided primarily by ethical values and emotional resonance, perceive animal welfare not merely as an added feature but as an intrinsic value. They firmly believe that purchasing such products is a direct reflection of their moral compass.

Interestingly, students who place an equivalent emphasis on both quality and emotion displayed the lowest WTP, with a figure of CNY 3.14, along with a broader standard deviation. This WTP does not neatly fit between the quality-centric and emotion-driven cohorts. One compelling explanation is their diminished price sensitivity (*β* = −0.315) relative to their counterparts. Such students are possibly influenced by a broader array of factors in their purchasing decisions, encompassing aspects like brand reputation and promotional tactics. These additional considerations may dilute their enthusiasm for directly supporting animal welfare when juxtaposed against the other two groups.

Advanced statistical scrutiny validated significant disparities in the premium WTP for animal welfare-labeled milk across the three student groups, with these differences being statistically significant at the 1% level. This revelation bears significant implications for market strategists seeking to finely calibrate their outreach to university students, tailored to their unique consumption propensities.

### 3.4. Sociodemographic Determinants of Purchase Intentions for Animal Welfare Milk

**Figure 4:**
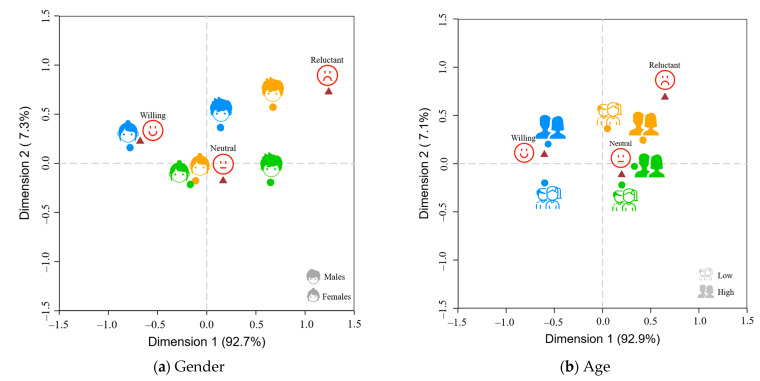

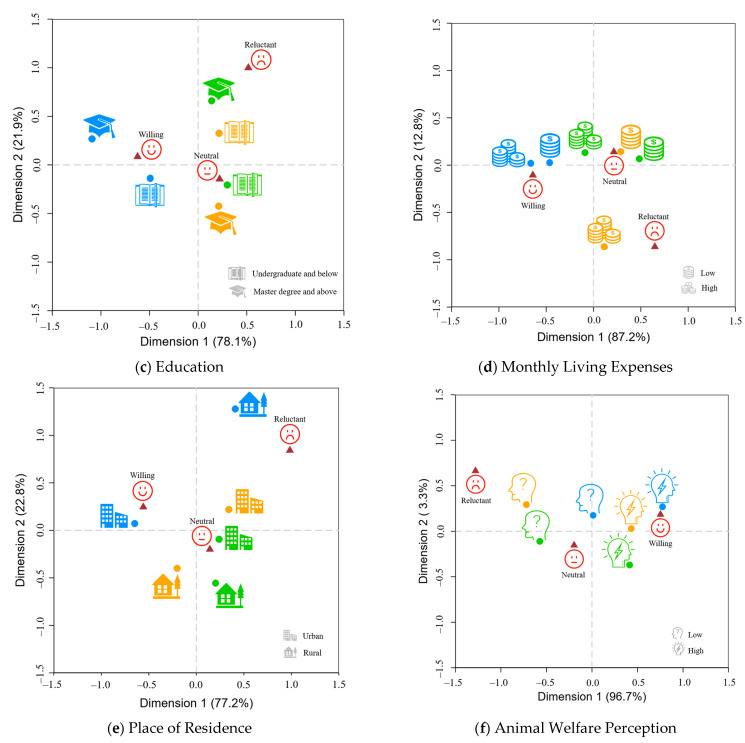
Interplay between sociodemographic traits and purchase intentions for animal welfare milk; ● symbolizes the Quality–Oriented group, ● denotes the Quality–Emotion Balanced group, and ● indicates the Emotionally Intuitive group.

We used the correspondence analysis method to identify demographic variations in purchasing inclinations for animal welfare-certified milk and to pinpoint the ideal consumer segment. We assessed purchase intentions by posing the question, “Would you be inclined to purchase milk bearing an animal welfare certification if it were available?” Responses were gauged on a 5-point Likert scale, with “1” representing “Definitely not” and “5” signifying “Definitely yes”. We stratified purchase intentions into three distinct tiers: “Reluctant to Buy” (scores 1–2), “Neutral” (score 3), and “Willing to Buy “ (scores 4–5). Data considered for visualization in Figure 4 met the criteria of independence at the 1% significance level. Importantly, closer point distances indicate a stronger association, reflecting characteristic agreement.

From the data, university students demonstrating a heightened purchase intent are predominantly female, older in age, hold an undergraduate degree or lower, have a superior monthly expenditure, are long-term urban dwellers, and exhibit robust animal welfare cognizance. Probing further, the “Willing to Buy” cohort can be more narrowly defined, whereas the “Reluctant to Buy” cluster appeared more amorphous. Interestingly, the “Emotionally Intuitive” segment, showing a strong preference for animal welfare certification, was closely tied to the “Willing to Buy” stance. Conversely, the “Quality–Oriented” demographic, exhibiting tepid enthusiasm for animal welfare milk, frequently aligned with the “Reluctant to Buy” category. These observations suggest that campaigns promoting animal welfare milk in the university marketplace should prioritize emotionally attuned consumers. In contrast, those prioritizing quality might remain more reticent. Consequently, emotive elements are paramount in shaping university students’ valuation of animal welfare-certified milk.

## 4. Discussion and Implications

### 4.1. Discussion

As highlighted in our introduction, the emphasis on animal welfare has grown substantially in recent years, both globally and in China. Our study aims to highlight its importance in the advancement of the livestock sector. Given the increasing focus on sustainability and animal rights, there is a growing demand for products that prioritize animal welfare [11,12,32]. Given the omnipresence of milk in everyday consumption, its intersection with animal welfare becomes a prescient issue. Building on the significance of milk as a symbol of nutrition for the younger generation, we sought to understand the consumption inclinations of university students, who are likely to become key supporters and promoters of animal welfare [5,15]. This study focuses on university students from Guangdong Province, China, using choice experiments to understand their preferences for milk associated with animal welfare and the reasons behind those preferences.

Our findings resonate with those of Cornish et al., (2020) [8] in their study on young Australian consumers, particularly in highlighting the pronounced inclination and awareness of animal welfare within the 18–29 age group. However, unlike the broader Australian demographic, our research specifically zooms in on Chinese university students, offering unique insights into this specific demographic. This positive inclination might stem from their enriched academic exposures, receptiveness to modern paradigms, and their active immersion in the digital sphere and social media landscapes. Broader consumer studies (spanning ages 18–65 and beyond) have corroborated the conclusion that females and individuals with augmented incomes exhibit an amplified willingness to shell out a premium for enhanced animal welfare [8,33], an observation resonating with our data from the student segment. Intriguingly, a significant portion of our student sample (35.58%) disclosed monthly expenditures of CNY 1500, with 25.66% reporting CNY 2000, suggesting a relatively comfortable financial milieu. This suggests that the evolving socioeconomic status in China is influencing these spending patterns, empowering the younger generation to make informed purchasing decisions based on ethics and values. Those with steeper outlays exhibit a heightened predisposition toward welfare-endorsed milk.

Additionally, our research reveals a range of preferences among students regarding animal welfare. While Wang and Gu (2014) [31] proposed that consumers’ rationale for championing animal welfare oscillates between product quality [34,35] and emotional alignment [36,37], our findings among university students shed light on the emerging importance of ethical consumption in China. Such importance is, to some extent, influenced by global trends, but is also inherently rooted in local dynamics [5]. This correlation between quality and emotion, as highlighted in our study, further finds empirical affirmation in the broader context of ethical consumer behavior. Furthermore, our research bolsters the assertions by Zingon et al., (2017) [3] and Vargas-Bello-Pérez et al. (2021) [15] that consumer judgments on animal welfare strike a nuanced equilibrium between quality and emotional dimensions. Segmenting students based on their orientations, we discerned three typologies: “Quality–Oriented”, “Emotionally Intuitive”, and “Quality–Emotion Balanced”. Collectively, these segments conveyed a shared endorsement of animal welfare, albeit with distinct willingness-to-pay and motivational contours.

Our study underscores the strong commitment of university students to animal welfare and offers valuable insights into future trends. While global narratives, such as those presented by Thorslund et al., (2016) [38] and Henriksen et al., (2022) [39], highlight an increased alignment with animal welfare among consumers—particularly in the European context—China faces its own unique challenges as an evolving market. One such pronounced challenge, which may be hinted at in global studies but is distinctly evident in our research, is the disparity between consumers’ perceptions and the actual living conditions of livestock in China. Only a mere 6.85% of our student cohort perceived suboptimal living conditions for dairy cows. However, research like that of Ding et al., (2022) [40] suggests a more concerning reality, emphasizing the gap due to informational shortcomings. This points to the pressing need for comprehensive, targeted educational interventions, tailored to enhancing students’ understanding of animal welfare.

Our study fills a significant gap by examining the perceptions of Chinese university students. Their attitudes set the stage for future inquiries and strategies in the context of a growing emphasis on ethically sourced products in the global market. However, certain caveats merit acknowledgment. The cultural and economic heterogeneity across China mandates a judicious extrapolation of insights gleaned from Guangdong Province students. Methodologically, while choice experiments furnish in-depth revelations on student predilections, the experimental configurations could subtly modulate the responses. However, we believe that our approach accurately captures genuine consumer orientations and sheds light on their decision-making processes. From a temporal vantage, given the dynamic landscape of animal welfare perceptions in China, our study offers a contemporaneous “capture” of prevailing attitudes—a valuable touchstone for subsequent inquiries. Future research endeavors might involve amplifying the sampling purview, finetuning the experimental paradigms, and chronicling the evolving narratives of animal welfare, thereby ensuring richer, more holistic insights. 

### 4.2. Implications

University students, recognized as future societal leaders, are instrumental in shaping the direction of animal welfare perceptions. Their perspectives provide not only a glimpse into potential societal shifts but also a basis for offering actionable guidance for both governmental and corporate entities.

Firstly, we consider the cultivation of animal welfare awareness through education. Recognizing the influential position that university students will soon occupy in society, it becomes vital to nurture a profound understanding of animal welfare within them. This can be achieved through the introduction of animal welfare-centric courses and by forging collaborations with international bodies to ensure an alignment with global best practices. Immersive experiences, like engagements with animal protection agencies or gaining insights into the livestock industry, can further solidify their understanding. On campuses, the establishment of animal welfare societies and the hosting of related events can offer students platforms to express, share, and implement their insights. Furthermore, integrating cultural initiatives, such as art exhibitions themed around animal welfare, can cultivate a more compassionate academic environment.

Secondly, we consider shaping an animal welfare-aligned market. It is crucial to establish a marketplace that genuinely emphasizes animal welfare. This entails prioritizing both product research and ethically driven innovation. Businesses should look beyond mere profitability, focusing also on ethical considerations in product development. Transparent product labeling indicating a commitment to animal welfare standards is paramount. This dedication should extend beyond the product itself, encompassing the entire supply chain, from sourcing raw materials to the final processing stages. Through periodic market assessments, companies can better align their offerings with the evolving expectations and preferences of consumers.

Lastly, we consider fostering an animal welfare-conscious society. Building a societal atmosphere that resonates with the principles of animal welfare demands a comprehensive approach. Efforts from governmental and institutional bodies should prioritize public outreach and education, aiming to deepen understanding and elicit widespread support for animal welfare. From a legislative perspective, the enactment of robust laws and policies that champion these values is essential. Collaborative initiatives with NGOs, especially those specializing in animal welfare, can play a pivotal role in driving awareness and engagement across the broader public. In the corporate realm, brands need to ensure that their commitment to animal welfare is both genuine and visible. Moreover, incorporating animal welfare education across all educational tiers, from primary to tertiary levels, will ensure these values are instilled early and reinforced consistently.

## 5. Conclusions

In contemporary consumer markets, a simple bottle of milk transcends its basic nutritional value, reflecting broader concerns about animal welfare. In this light, our research directed its lens toward Chinese university students, aiming to understand their consumption dispositions and preferences for milk products adorned with animal welfare certifications. Using the discrete choice experiment approach, we discerned a marked preference among this demographic for milk products with animal welfare certification. When unpacking the consumption determinants, 48.77% of students emerged as the “Quality–Emotion Balanced” segment, followed by the “Emotionally Intuitive” segment at 30.67%, and the “Quality–Oriented” segment encompassing 20.55%. Across the board, these segments demonstrated a favorable bias toward animal welfare, with the “Emotionally Intuitive” segment standing out prominently. These findings underscore that while considerations of quality and ethical alignment shape students’ dairy choices, personal consumption motivations wield significant influence. Further, our empirical assessment underscored variations in the premium that different segments were prepared to offer.

Our inquiry offers insights into Chinese university students’ dispositions and preferences toward dairy products associated with animal welfare, concurrently casting a light on overarching market dynamics and strategies. The consumer ethos exhibited by university students, to a significant degree, promises to sculpt the trajectory of the broader market landscape. Consequently, understanding their preferences is not only pivotal for China’s animal welfare market but could also offer valuable strategic perspectives for international markets. It is crucial to recognize the study’s inherent scope limitations, primarily its circumscribed focus on Chinese university students, which might temper the universality of the insights. With global interconnectivity intensifying, subsequent research could embrace broader student cohorts across diverse global regions, helping to compare consumption behaviors and market details. Such comparative endeavors would not only render a richer global panorama but would also accentuate the central role that university students inhabit in steering consumer trends. Beyond this, future investigations could venture beyond the university student demographic, expanding the investigative ambit to incorporate a wider consumer spectrum, thereby offering a more nuanced market perspective. Our work presents invaluable reflections for academics, policymakers, and industry stakeholders, underscoring its transnational pertinence. We hope that our contributions catalyze further advancements and innovation in the animal welfare marketplace.

## Figures and Tables

**Table 1:** Attributes and their levels for the choice experiment.

Attributes	Description	Levels
Brand	Includes domestic and international brands	Domestic, International
Label	Indicates whether the milk meets animal welfare or organic certification standards	No label, Organic, Animal welfare
Protein	Protein content per 100 mL of milk (g)	3.2, 3.6, 4.0
Shelf Life	Shelf life of packaged food under specified storage conditions (months)	1, 3, 5
Price	Price per 250 mL milk bottle (CNY/bottle)	2.8, 4.8, 6.8, 8.8

**Table 2:** Detailed demographic and consumption characteristics of respondents.

Characteristics	Value	Characteristics	Value
Gender (%)		Milk Purchasing Channel (%)	
Male	41.00	Online	58.69
Female	59.00	Offline	41.31
Age		Years of Milk Consumption (%)	
Mean (S.D.)	20.82 (2.15)	≤1	7.98
Education Level (%)		1–3	11.35
Undergraduate	85.17	4–6	17.79
Graduate	14.83	7–10	13.70
Place of Residence (%)		≥11	49.18
Urban	85.79	BMI (kg/m^2^)(%)	
Rural	14.21	≤18.50	17.79
Monthly Living Expense		18.50–23.99	65.03
Mean (S.D.)	1732.34 (693.86)	≥24.00	17.18

Note: The provided BMI classification adheres to the guidelines set forth by the National Health Commission of the People’s Republic of China. The delineation criteria are as follows: underweight for a BMI of less than 18.5 kg/m^2^, normal weight ranges between 18.5 and 23.9 kg/m^2^, overweight lies between 24.0 and 27.9 kg/m^2^, and obesity is categorized as a BMI of 28 kg/m^2^ or above.

**Table 3:** Students’ perceptions of animal welfare.

Item (Statement)	Mean	Standard Deviation	Reliability	Overall Reliability
Since farm animals will ultimately be slaughtered for consumption, their welfare does not matter.	2.374	0.965	0.812	
Human welfare has yet to be met, and it is not time to consider animal welfare.	2.634	1.089	0.785	0.850
The cost of welfare-oriented farming is too high and not suitable for our country’s reality.	2.876	0.979	0.790	
Some welfare farming practices are merely due to farmers’ curiosity and novelty, or for commercial selling points.	2.808	1.042	0.846	

**Table 4:** Cognition of farm animal welfare milk.

Item (Statement)	Mean	Standard Deviation	Reliability	Overall Reliability
The taste of animal welfare milk is better.	3.247	0.686	0.897	0.909
Animal welfare milk is safer.	3.365	0.746	0.875	
Animal welfare milk is healthier.	3.396	0.769	0.874	
Drinking animal welfare milk makes me feel better.	3.374	0.825	0.887	
Purchasing animal welfare milk expresses my concern for farm animals.	3.454	0.848	0.907	

**Table 5:** Regression results for preferences toward animal welfare milk.

Attributes	Full Sample	Agriculture	Science	Engineering	Humanities
Mean					
Price	−0.344 ***	−0.349 ***	−0.311 ***	−0.314 ***	−0.406 ***
	(0.013)	(0.028)	(0.027)	(0.025)	(0.030)
Import	−0.060	0.052	−0.117	0.001	−0.136
(Baseline: Domestic)	(0.044)	(0.084)	(0.088)	(0.091)	(0.094)
Organic	1.377 ***	1.277 ***	1.377 ***	1.298 ***	1.533 ***
(Baseline: No Label)	(0.062)	(0.131)	(0.126)	(0.120)	(0.139)
Animal Welfare	1.237 ***	1.185 ***	1.228 ***	1.059 ***	1.455 ***
(Baseline: No Label)	(0.061)	(0.129)	(0.124)	(0.112)	(0.138)
Protein	0.466 ***	0.385 **	0.381 ***	0.599 ***	0.262 *
	(0.072)	(0.153)	(0.145)	(0.143)	(0.156)
Shelf Life	0.024 *	0.013	0.008	0.029	0.044
	(0.013)	(0.027)	(0.027)	(0.025)	(0.029)
No Purchase	−1.963 ***	−2.471 ***	−1.672 ***	−1.415 **	−2.713 ***
	(0.291)	(0.625)	(0.568)	(0.562)	(0.633)
Standard Deviation					
Import	0.687 ***	−0.418 **	0.601 ***	0.863 ***	0.626 ***
(Baseline: Domestic)	(0.064)	(0.177)	(0.148)	(0.128)	(0.139)
Organic	0.705 ***	0.669 ***	0.738 ***	0.761 ***	0.677 ***
(Baseline: No Label)	(0.087)	(0.197)	(0.174)	(0.164)	(0.183)
Animal Welfare	0.536 ***	0.525 *	0.457 *	0.518 **	0.550 ***
(Baseline: No Label)	(0.106)	(0.302)	(0.262)	(0.219)	(0.212)
Protein	0.521 ***	0.751 ***	0.517 ***	0.605 ***	0.458 ***
	(0.040)	(0.086)	(0.069)	(0.092)	(0.117)
Shelf Life	−0.007	0.039	0.054	−0.005	0.101 *
	(0.027)	(0.044)	(0.050)	(0.051)	(0.054)
No Purchase	−1.438 ***	0.485	0.328	0.328	−1.058 *
	(0.172)	(0.527)	(0.343)	(0.811)	(0.552)
Model Fit					
LR chi2	792.23	240.74	142.83	234.47	104.55
Log likelihood	−4452.3548	−955.8887	−1062.6250	−1226.6594	−942.8218
AIC	8930.710	1937.777	2151.250	2479.319	1911.644
BIC	9031.796	2019.110	2233.118	2563.577	1993.037
Observations	17,604	3852	4014	4824	3870

Note: Levels of significance have been demarcated as ***, **, and * to represent the 1%, 5%, and 10% thresholds, respectively. Values in parentheses are standard errors.

**Table 6:** Segmented analysis of preferences for animal welfare milk.

Attributes	Quality–Oriented	Quality–Emotion Balanced	Emotionally Intuitive
Mean			
Price	−0.347 ***	−0.315 ***	−0.414 ***
	(0.027)	(0.019)	(0.027)
Import (Baseline: Domestic)	−0.087	−0.043	−0.029
	(0.091)	(0.065)	(0.083)
Organic (Baseline: No Label)	1.484 ***	1.268 ***	1.607 ***
	(0.138)	(0.093)	(0.113)
Animal Welfare	1.283 ***	1.021 ***	1.646 ***
(Baseline: No Label)	(0.128)	(0.088)	(0.122)
Protein	0.580 ***	0.483 ***	0.355 ***
	(0.153)	(0.108)	(0.128)
Shelf Life	0.028	0.018	0.037
	(0.029)	(0.019)	(0.024)
No Purchase	−1.294 **	−2.237 ***	−1.966 ***
	(0.631)	(0.436)	(0.519)
Standard Deviation			
Import (Baseline: Domestic)	0.511 ***	0.757 ***	0.815 ***
	(0.145)	(0.095)	(0.117)
Organic (Baseline: No Label)	0.766 ***	0.870 ***	−0.365 *
	(0.176)	(0.130)	(0.188)
Animal Welfare	0.089	0.705 ***	0.645 ***
(Baseline: No Label)	(0.309)	(0.141)	(0.176)
Protein	0.346 ***	0.756 ***	0.089
	(0.118)	(0.060)	(0.094)
Shelf Life	0.007	0.004	0.023
	(0.097)	(0.037)	(0.042)
No Purchase	1.622 ***	0.361	−1.629 ***
	(0.396)	(0.287)	(0.214)
Model Fit			
LR chi2	151.15	545.07	129.25
Log likelihood	−917.788	−2171.489	−1323.818
AIC	1861.576	4368.978	2673.636
BIC	1942.094	4460.730	2759.360
Observations	3618	8586	5400

Note: Levels of significance have been demarcated as ***, **, and * to represent the 1%, 5%, and 10% thresholds, respectively. Values in parentheses are standard errors.

## Data Availability

The datasets generated and/or analyzed during the current study are available from the corresponding author on reasonable request.
